# Effects of the number of markers per haplotype and clustering of haplotypes on the accuracy of QTL mapping and prediction of genomic breeding values

**DOI:** 10.1186/1297-9686-41-11

**Published:** 2009-01-15

**Authors:** Mario PL Calus, Theo HE Meuwissen, Jack J Windig, Egbert F Knol, Chris Schrooten, Addie LJ Vereijken, Roel F Veerkamp

**Affiliations:** 1 Animal Breeding and Genomics Centre, Animal Sciences Group, Wageningen University and Research Centre, P.O. Box 65, 8200 AB Lelystad, The Netherlands; 2 University of Life Sciences, Department of Animal and Aquacultural Sciences, Ås, Norway; 3 IPG, Beuningen, The Netherlands; 4 CRV, Arnhem, The Netherlands; 5 Hendrix Genetics B.V., Boxmeer, The Netherlands

## Abstract

The aim of this paper was to compare the effect of haplotype definition on the precision of QTL-mapping and on the accuracy of predicted genomic breeding values. In a multiple QTL model using identity-by-descent (IBD) probabilities between haplotypes, various haplotype definitions were tested *i.e*. including 2, 6, 12 or 20 marker alleles and clustering base haplotypes related with an IBD probability of > 0.55, 0.75 or 0.95. Simulated data contained 1100 animals with known genotypes and phenotypes and 1000 animals with known genotypes and unknown phenotypes. Genomes comprising 3 Morgan were simulated and contained 74 polymorphic QTL and 383 polymorphic SNP markers with an average r^2 ^value of 0.14 between adjacent markers. The total number of haplotypes decreased up to 50% when the window size was increased from two to 20 markers and decreased by at least 50% when haplotypes related with an IBD probability of > 0.55 instead of > 0.95 were clustered. An intermediate window size led to more precise QTL mapping. Window size and clustering had a limited effect on the accuracy of predicted total breeding values, ranging from 0.79 to 0.81. Our conclusion is that different optimal window sizes should be used in QTL-mapping versus genome-wide breeding value prediction.

## Introduction

The use of genome-wide dense marker maps in animal breeding is becoming more common for both genome-wide breeding value prediction and QTL detection. In genome-wide breeding value prediction, the simplest model assumes that each allele of a marker locus has an effect on the trait of interest, *i.e*. that a simple regression on single or multiple SNP markers can be used as predictive model for the breeding value. Alternatively, haplotypes can be constructed using marker alleles of two or more loci on the same chromosome. In this type of analysis, haplotypes are associated to the phenotypic values, and the summation of all haplotype effects gives the genomic breeding value of an animal. Using the haplotype approach, different assumptions can be made about relationships between haplotypes. For example, one option is to assume that a specific haplotype has a one to one relation with the same QTL allele independently of the individual that carries the haplotype [*e.g*. [1]]. Alternatively, identical-by-descent probabilities (IBD) between marker haplotypes can be used to allow for non zero relationships between haplotypes and relationships less than unity between two identical marker haplotypes carried by different individuals [*e.g*. [2]]. For example, a smaller than unity IBD probability between identical haplotypes can be explained by the fact that marker alleles are inherited from different ancestors. Using both haplotypes and IBD probabilities in the analysis has the advantage that not only population-wide linkage disequilibrium between markers and QTL, but also within family linkage disequilibrium between markers and QTL is taken into account. These different models have proven to be important for the prediction of genomic breeding values. Including multiple markers per haplotype and IBD information, with a moderate marker density generally yields more accurate results than models that include haplotypes based on two marker alleles but not relations between haplotypes, or models that include marker alleles instead of haplotypes [3].

Also, in applications for QTL fine-mapping, the differences in models have been investigated. It has been shown that using a reduced number of marker alleles in a haplotype based IBD method, yields higher mapping accuracy than using all available marker alleles in haplotypes [4]. An important question is what is the effect of the number of included markers in haplotypes on the accuracy of predicted breeding values. Furthermore, in contrast to the results obtained by predicting breeding values, it has been shown that for QTL mapping regression on single marker alleles can compete with haplotype based methods using IBD [5]. One factor that might play an important role in this comparison is the number of effects that needs to be estimated in the model. It has been shown that a model including both haplotypes based on multiple markers and relationships between them, yields ~25 to 500 times as many effects that need to be estimated as a model based on single marker alleles [3]. Reducing the number of haplotypes reduces the degrees of freedom used in the model, which produces increased power in association studies [6]. If reducing the number of effects in the model is an important factor for accurately estimating QTL, then reducing the number of haplotypes by clustering haplotypes that are strongly related to each other [7,6], might be another option to improve the accuracy of QTL detection.

The aim of this paper was to investigate the effect of haplotype definition on the accuracy of predicted breeding values for genomic selection and on the precision of QTL mapping, given a moderately dense marker map and using simulated data. Various haplotype definitions were tested in the IBD-based multiple QTL model by changing the number of surrounding marker alleles used per haplotype and the degree of haplotypes clustering together depending on their IBD probability (> 0.55, 0.75 and 0.95).

## Methods

### Simulation

For each replicate, an effective population size of 100 animals was simulated for 1,000 generations. Each next generation was formed by generating 100 offspring (50 males and 50 females), their parents selected at random from the current generation.

To reduce calculation time, the simulated genomes comprised three chromosomes of 1 Morgan each. The positions of 7 500 QTL and 50 000 marker loci were simulated randomly across the genome. In the first generation, all QTL and marker loci had an allele coded as 1. The probability of having a recombination between two adjacent loci on the same chromosome was calculated using Haldane's mapping function based on the distance between the loci. In generation 1 through 1000, on average 50 markers and 7.5 QTL mutations per generation were simulated, yielding mutated alleles coded as 2. Each locus had one mutation during the 1000 generations. The initial numbers of marker and QTL loci were determined based on the number of loci that were still polymorphic in generation 1000 in preliminary analyses, targeting respectively ~400 polymorphic SNP and 80 polymorphic QTL on the 3 Morgan genomes. Because 1000 generations of random mating were simulated, linkage disequilibrium (LD) could arise between marker and QTL loci due to random genetic drift, as shown in other studies [3,8,1,10]. This arisen LD between marker and QTL loci provided associations between QTL loci and marker haplotypes as a result of population history [11].

All original QTL alleles were assumed to have no influence on the considered trait. All mutated QTL alleles received an effect drawn from a gamma distribution (with a shape parameter of 0.4 and scale parameter of 1.0), with an equal chance of being positive or negative according to Meuwissen *et al*. [1]. The gamma distribution ensured that a large number of QTL had small effects, while a small number of QTL had large effects and explained much of the genetic variance, as shown for QTL in livestock [12,13]. Three additional generations (1001 to 1003) were simulated in which no mutations occurred. The simulated additive genetic variance at each locus *i *($\sigma_{gi}^{2}$) was calculated using allele frequencies calculated from those three additional generations, using the formula $\sigma_{gi}^{2}$ = *2p(1-p)a*^2 ^[14], where *p *is the allele frequency of one of both alleles at a QTL locus, and *a *is the allele substitution effect. The total simulated genetic variance ($\sigma_{g}^{2}$) was obtained by summing up the variance across all QTL loci, assuming no correlation between QTL. To obtain a heritability of 0.50, the residuals were drawn from a random distribution *N*(0, $\sigma_{g}^{2}$). All animals in generations 1001 and 1002 received one phenotypic record, obtained by adding a random residual to the true breeding value of the animals. All phenotypic records were scaled such that the phenotypic variance was 1.0. Generation 1001 comprised 100 animals. Generation 1002 comprised 1000 animals, meaning that animals of generation 1001 on average had 20 offspring, whereas parents of previous generations on average had two offspring. The generation 1002 produced one more generation of 1000 offspring. Thus, 1100 animals (generations 1001 and 1002) with known phenotypes and genotypes were simulated, as well as 1000 juvenile animals with unknown phenotypes and known genotypes (generation 1003).

### Analysis

The general model to estimate the haplotype effects at *nloc *putative QTL loci in the simulated dataset was:

$$y_{i}=\mu +animal_{i}+\sum_{j=1}^{nloc}\left(q_{ij1}+q_{ij2}\right)v_{j}+e_{i}$$

where *y*_*i *_is the phenotypic record of animal *i*, *μ *is the average phenotypic performance, *animal*_*i *_is the random polygenic effect for animal *i*, *v*_*j *_is the direction of the haplotype effects at a putative QTL position *j*, *q*_*ij*1 _(*q*_*ij*2_) is the size of the QTL effect for the paternal (maternal) haplotype of animal *i *at locus *j *(of *nloc *putative QTL loci) of animal *i*, and *e*_*i *_is a random residual for animal *i*. Gibbs sampling was used for the analysis, using a Gauss Seidel iteration on data solving scheme together with simultaneous variance component estimation, as recommended by Legarra and Misztal [15] for genome-wide breeding value prediction. The Gibbs sampling process included sampling of the presence of a QTL at each considered putative QTL position. Putative QTL loci were considered at the midpoint of each pair of adjacent markers on the same chromosome, following Meuwissen and Goddard [2]. Hence, when *m *markers were simulated across the three chromosomes, *m*-3 putative QTL positions were considered. For each putative QTL locus, haplotypes were defined using different numbers of surrounding markers, as explained in the next section.

For simplicity, linkage phases of marker alleles were assumed to be known without error. Between all the haplotypes at the same locus, the probability of being IBD was calculated, combining linkage disequilibrium and linkage analysis information. The IBD probabilities between haplotypes of the first generation of genotyped animals, were predicted using a simplified coalescence process, with the assumptions that 100 generations were between the current and base population and that the effective population size during those 100 generations was 100. The calculated IBD matrix for the base haplotypes was inverted. Whenever one of the eigenvalues of the matrix after clustering (which is described in the next section) was smaller than 0.0, *i.e*. when the matrix was not positive definite, the matrix was bended by adding *|min_eigenval| *+ 0.01 to all the diagonal elements, where *|min_eigenval| *is the absolute value of the lowest (negative) eigenvalue. Haplotypes of animals in later generations were added to the inverted IBD matrices using the recursive formulas as described by Fernando and Grossman [16]. A full description of the method to predict the IBD probabilities is given by Meuwissen and Goddard [2]. The IBD-matrix was used to model the covariances between haplotypes.

The covariance among polygenic effects was estimated as A × $\sigma_{G}^{2}$, where A is the additive genetic relationship matrix based on the pedigree of the last four generations of animals and $\sigma_{G}^{2}$ is the polygenic variance. The estimated haplotype variance at each locus was calculated as H × ${\hat{\sigma }}_{h}^{2}$, where H is the heterozygosity of clustered haplotypes in the analysed population and ${\hat{\sigma }}_{h}^{2}$ is the estimated posterior haplotype variance for the base population. ${\hat{\sigma }}_{h}^{2}$ was considered to be equal to the estimated variance of *v*_*j *_× *q*_.*j*._. The (co)variance of haplotypes at locus *j *(*q*_.*j*._) was modelled by the IBD matrix for locus *j*. Since diagonal elements of the IBD matrix have a value of 1.0, the model restricted *q*_.*j*. _to have a variance of 1 [17], and therefore ${\hat{\sigma }}_{h}^{2}$ was calculated as ${\hat{v}}_{j}^{2}$. The formula H × ${\hat{\sigma }}_{h}^{2}$ is analogous to the formula *2pqa*^2 ^where *2pq *is the heterozygosity at a biallelic locus and *a *is the allele substitution effect [14], and also analogous to the calculation of an additive genetic variance as (1-*F*) × ${\hat{\sigma }}_{a}^{2}$ where *F *is the inbreeding in the current population. In our situation, we assumed that animals were unrelated in the considered base population (100 generations ago), meaning that in the base population the IBD probability between paternal and maternal haplotypes at a locus was 0.0. The heterozygosity at a locus in the analysed population was estimated as follows:

1) the probability that an animal was heterozygous at a locus, was equal to the probability that the paternal and maternal alleles were non-IBD

2) the heterozygosity per locus was calculated as the average probability (across animals) that an animal was heterozygous at this locus.

The presence of a QTL at a putative QTL locus *j *was sampled from a Bernoulli distribution: $\frac{\text{P}({\hat{v}}_{j}|{\hat{\sigma }}_{\text{V}}^{\text{2}})\times {\mathrm{Pr}}_{\text{j}}}{\text{P}({\hat{v}}_{j}|{\hat{\sigma }}_{\text{V}}^{\text{2}})\times {\mathrm{Pr}}_{\text{j}}+\text{P}({\hat{v}}_{j}|{\hat{\sigma }}_{\text{V}}^{\text{2}}/100)\times (1-{\mathrm{Pr}}_{j})}$, where P(${\hat{v}}_{j}$ | ${\hat{\sigma }}_{\text{V}}^{\text{2}}$) is the probability of sampling ${\hat{v}}_{j}$ from N(0, ${\hat{\sigma }}_{\text{V}}^{\text{2}}$), ${\hat{\sigma }}_{\text{V}}^{\text{2}}$ is the variance of the direction vector at locus *j*, and Pr_*j *_is the prior probability of the presence of a QTL at putative QTL locus *j *[17]. It was assumed that from prior knowledge one QTL was expected per chromosome. Therefore, prior QTL probabilities were calculated as the distance between the two markers surrounding the putative QTL position *j*, divided by the total length of the chromosome. Initially, presence of a QTL was considered at each putative QTL position, *i.e*. all QTL indicators at the start of the analysis were considered to be 1. The average posterior QTL indicator at each locus, after the burn-in, was calculated to obtain the mean posterior QTL probability at each locus. The Gibbs sampler was run for 30 000 iterations, of which the first 3000 iterations were discarded as burn-in. The Gibbs sampler is described in more detail by Meuwissen and Goddard [17].

### Definition of windows and clustering of related haplotypes

Two types of haplotypes were considered: 1) the haplotypes of the first generation of genotyped animals (referred to as base haplotypes), and 2) the haplotypes of second and later generations of genotyped animals (referred to as non-base haplotypes).

**Figure 1:**
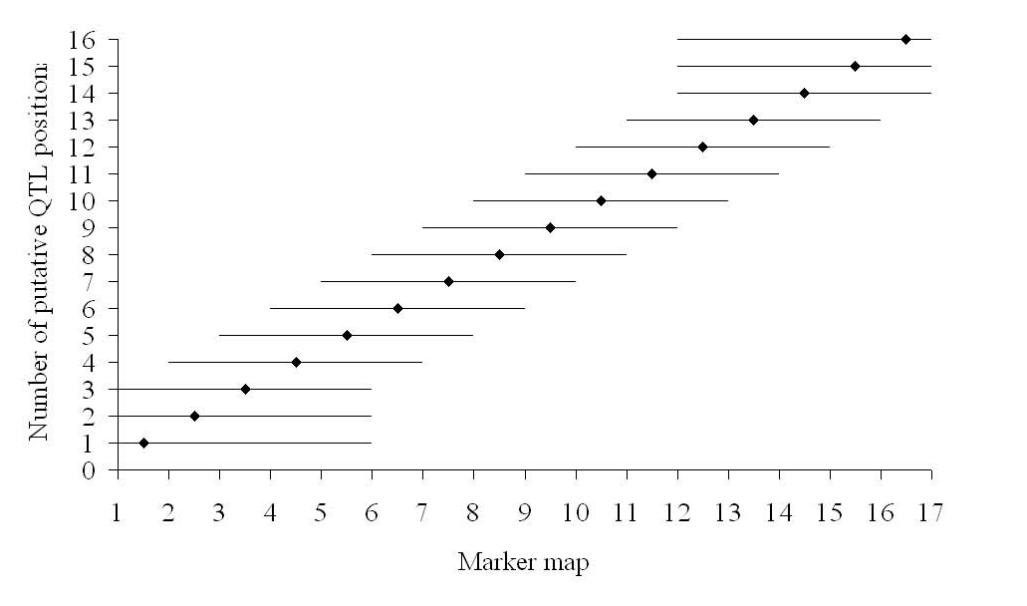
**The sliding window of six markers (--) given the different putative QTL positions (◆), on a chromosome with 17 equally spaced markers**.

Base haplotypes were formed based on a sliding window of 2, 6, 12 or 20 markers on the same chromosome, with the putative QTL position whenever possible between respectively the 1st and 2nd, 3rd and 4th, 6th and 7th, and 10th and 11th marker. When the number of markers was insufficient to the 'left' or the 'right' of a QTL position, more markers from the other side of the putative QTL position were arbitrarily added to ensure that the window contained the required number of markers. An example of a sliding window of six markers across a chromosome with 17 markers is given in Figure 1.

Each animal has two haplotypes at each locus, which implies that the maximum number of constructed haplotypes is twice the number of animals (*2n*). Initially, the IBD-probabilities between all possible pairs of the base haplotypes (*2n*_*b*_) were calculated, using the method described by Meuwissen and Goddard [17]. Those *2n*_*b *_base haplotypes were clustered, using a hierarchical clustering algorithm that involved the following steps:

1) identification of all pairs of base haplotypes with an IBD probability among them of >*limitIBD;*

2) clustering of all pairs of haplotypes identified in step 1, summing the mutual off-diagonal elements with other haplotypes and counting the number of haplotypes per formed cluster. Whenever one (or both) of the two haplotypes was already assigned to a cluster, the counts and sum of off-diagonal values of the clustered haplotypes were used instead of the values for the haplotype before clustering;

3) for each clustered haplotype, the summed off-diagonals were divided by the number of haplotypes in the cluster.

Effectively, when *nh *haplotypes were clustered to haplotype 1*, the IBD probability between for instance haplotype 1* and *k *was calculated as

$$P_{IBD}(1^{*},k)\sum_{i=1}^{nh}(P_{IBD}(i,k))/nh.$$

The considered values for *limitIBD *were 0.55, 0.75 and 0.95 for pairs of base haplotypes. Pairs of haplotypes, of which at least one haplotype was not a base haplotype, were clustered using the same steps as for base haplotypes, but only considering a value for *limitIBD *of 0.95.

### Evaluation of analyses

Each simulated dataset and model analysis was replicated ten times for *limitIBD *of 0.75 and 0.95 and all four window sizes, while in total 52 replicates were considered for *limitIBD *of 0.55 and all four window sizes. More replicates were considered only for *limitIBD *of 0.55, to enable more precise assessment of the differences in the prediction of the QTL position for different window sizes, since the first ten replicates showed that different values for *limitIBD *for the base haplotypes hardly influenced the predicted QTL position. Accuracies of total breeding values were calculated as the average correlation across replicates between simulated and predicted breeding values of animals without phenotypic information. The bias of the predicted total breeding values of juvenile animals was also evaluated, by plotting the difference between simulated and predicted total breeding values for the juvenile animals minus the estimated phenotypic mean in the model, against the true breeding values corrected for the true mean.

To determine the capacity of the applied methods to position QTL precisely using different window sizes and values for *limitIBD*, we determined the frequency of situations in which posterior evidence for a QTL was found at or nearby simulated QTL positions. To achieve this, for each analysis, marker intervals with a simulated QTL that explained at least 5% or between 2 and 5% of the phenotypic variance were identified. For those marker intervals and ten surrounding intervals, the posterior probability was averaged across replicates.

## Results

The 52 simulated replicates had on average 74 polymorphic QTL loci and 383 polymorphic marker loci in generations 1001, 1002 and 1003, resulting in an average r^2 ^value (measure for LD; [18]) between adjacent markers of 0.14. Average minor allele frequencies were 0.167 for the QTL and 0.176 for the markers.

### Average numbers of (base) haplotypes

The effect of different degrees of haplotype clustering based on IBD and the used number of surrounding markers (windows size) in haplotypes on the average number of haplotypes in the base population and non-base haplotypes are shown in Table 1. Increasing window sizes and lowering *limitIBD *for clustering haplotypes decreased the number of base and non-base haplotypes. Increasing the numbers of base haplotypes decreased the number of additional non-base haplotypes. When the window size was increased from two to 20 markers, the reduction in the total number of haplotypes ranged from 36 to 50% depending on *limitIBD*. The total number of haplotypes at a clustering limit of IBD < 0.55 was less than half the number of haplotypes at a clustering limit of 0.95. It should be noted that initially 4200 haplotypes were defined per locus, *i.e*. 2 haplotypes * 2100 animals. Therefore, applying the standard clustering limit of 0.95 already decreased the number of haplotypes to 10% of the initial number.

**Table 1:** Average number of base, non-base and total haplotypes per locus across replicates, at different limits of clustering of base haplotypes and different window sizes

Haplotypes	Window size	Clustering limit base haplotypes		
		
		0.55	0.75	0.95
**Base**	2	48.2	124.1	172.4
	6	7.6	35.8	101.9
	12	6.5	15.5	45.2
	20	6.2	14.0	33.7

**Non base**	2	170.5	288.6	338.5
	6	114.6	202.4	291.4
	12	126.8	186.2	253.9
	20	133.9	194.0	251.7

**Total**	2	218.7	412.7	510.9
	6	122.2	238.2	393.3
	12	133.3	201.7	299.1
	20	140.1	208.0	285.4

### Accuracy and bias of predicted total breeding values

Accuracies of total predicted breeding values of animals with phenotypes were all in the range of 0.88 to 0.89 (results not shown). Accuracies of total predicted breeding values of juvenile animals were quite similar for different clustering limits and window sizes (Table 2). However, the accuracies were about 0.02 lower at a value for *limitIBD *of 0.55 compared to a value of 0.95 at window sizes of 6, 12 and 20. Accuracies at window sizes of 20 compared to two were 0.01 and 0.02 higher at clustering *limitIBD *0.75 and 0.95, respectively. In the additional 42 replicates at a *limitIBD *of 0.55, the differences in accuracies between different window sizes (results not shown) were similar to those in the first ten replicates.

**Table 2:** Accuracies of total predicted breeding values of juvenile animals averaged across 10 replicates

	Clustering limit base haplotypes		
**Window size**	0.55	0.75	0.95
2	0.794	0.791	0.792
6	0.792	0.806	0.813
12	0.785	0.800	0.813
20	0.794	0.805	0.813

Standard errors ranged from 0.0029 to 0.0032

**Figure 2:**
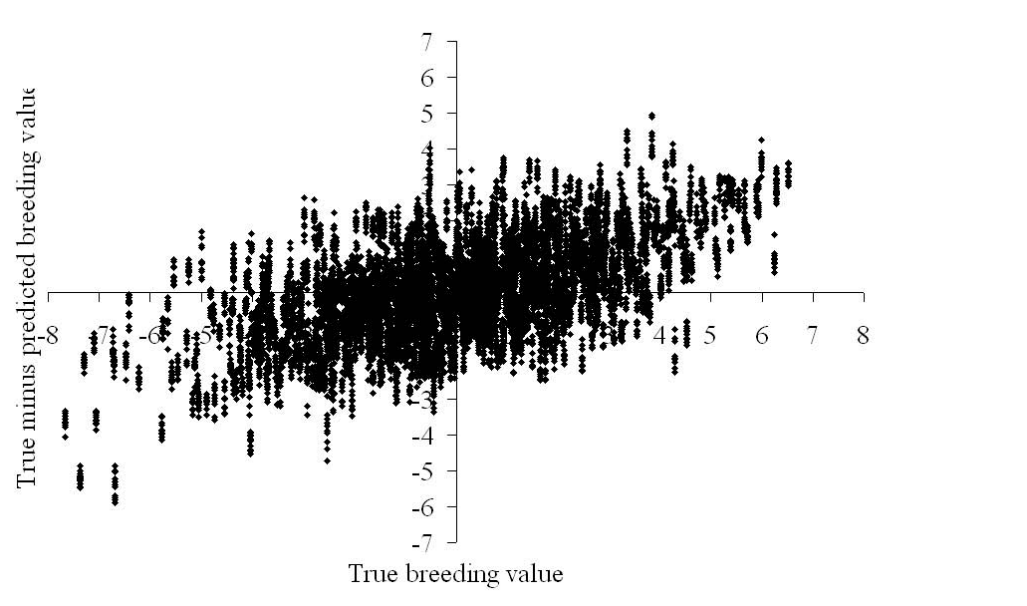
**Difference between true (corrected for the true mean) and predicted (corrected for the estimated mean) total breeding values plotted against the true breeding values of all juvenile animals for all 12 analyses in replicate 1**.

The bias of the predicted breeding values did not show apparent differences at different window sizes and values for *limitIBD *(results not shown). Bias of the predicted breeding value for the juvenile animals, calculated as the differences between simulated and predicted true breeding values, tended to be higher at more extreme true breeding values (Fig. 2). The bias showed that predicted breeding values were generally closer to the mean than true breeding values (Fig. 2), indicating that the estimated genetic variance was lower than the simulated genetic variance and breeding values were underestimated.

### Estimated variance components

Estimated haplotype, polygenic, total genetic and residual variances are shown in Table 3. Estimated haplotype variance was hardly affected by differences in window size or *limitIBD*. Surprisingly, the estimated polygenic variance increased with decreasing value of *limitIBD *and increased with increasing window size. For all the scenarios, the total genetic variance was underestimated and the estimated residual variance was close to the simulated value.

**Table 3:** Estimated haplotype, polygenic, total genetic and residual variances and heritabilities

Clustering limit base haplotypes	Window size	Haplotype variance	Polygenic variance	**Total genetic variance**^1^	**Residual variance**^1^
0.55	2	0.204	0.100	0.304	0.491
	6	0.186	0.149	0.335	0.498
	12	0.177	0.172	0.349	0.488
	20	0.184	0.169	0.353	0.485

0.75	2	0.195	0.073	0.268	0.476
	6	0.204	0.103	0.307	0.491
	12	0.195	0.124	0.319	0.490
	20	0.192	0.139	0.331	0.483

0.95	2	0.191	0.063	0.253	0.475
	6	0.201	0.064	0.265	0.483
	12	0.200	0.100	0.301	0.482
	20	0.198	0.104	0.302	0.486

^1 ^Standard errors ranged from 0.013 to 0.018 for the haplotype variance, from 0.010 to 0.030 for the polygenic variance, from 0.016 to 0.033 for the total genetic variance and from 0.010 to 0.015 for the residual variance

### Posterior QTL probabilities

**Figure 3:**
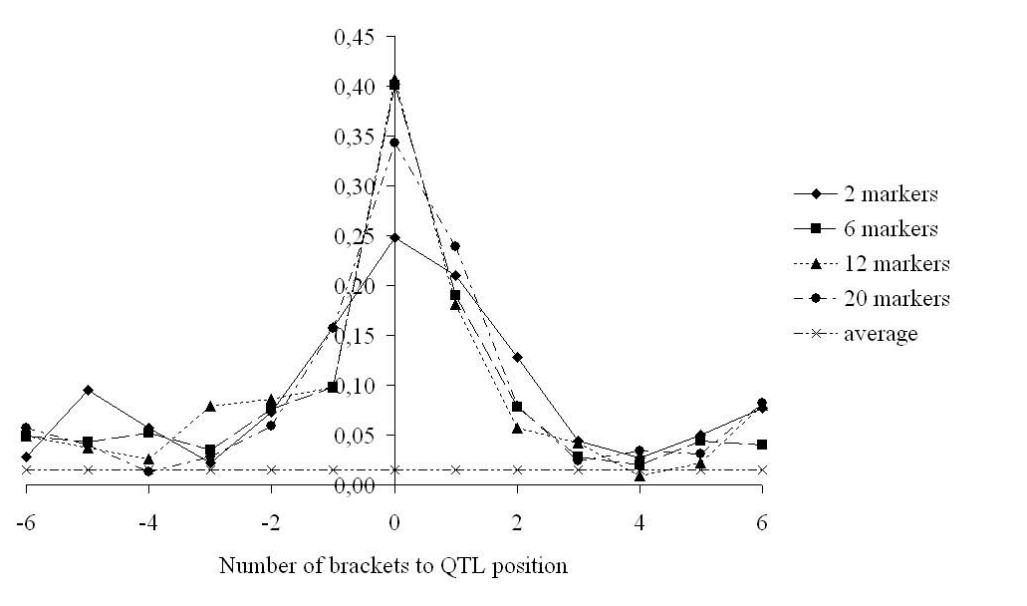
**Average posterior probabilities (across 80 replicates) of a fitted QTL in (neighbouring) brackets where a QTL was simulated with a variance > 0.05 σ_p_^2 ^for clustering of base haplotypes with a limit of 0.55, and window sizes of 2, 6, 12 and 20 markers, and on average across all brackets**. On average, per replicate there were 2.88 such simulated QTL

**Figure 4:**
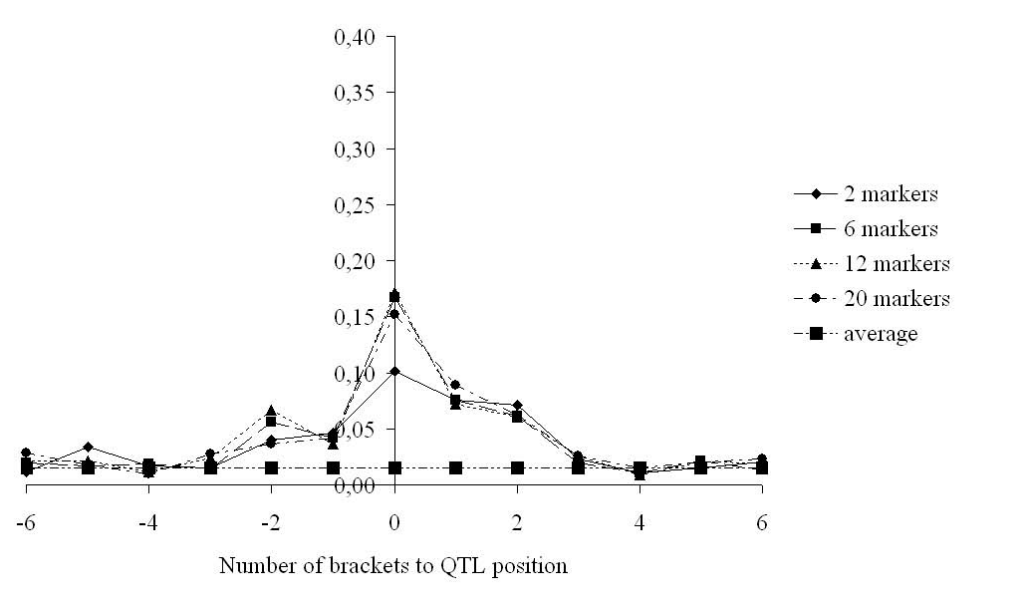
**Average posterior probabilities (across 80 replicates) of a fitted QTL in (neighbouring) brackets where a QTL was simulated with a variance > 0.02 σ_p_^2 ^and < 0.05 σ_p_^2 ^for clustering of base haplotypes with a limit of 0.55, and window sizes of 2, 6, 12 and 20 markers, and on average across all brackets**. On average, per replicate there were 3.21 such simulated QTL

In order to compare the capacity of the different scenarios to map QTL, we identified regions in which QTL were segregating that explained 5% (Fig. 3) or between 2 and 5% of the phenotypic variance (Fig. 4). For both groups of QTL, the scenario with a window size of 2 often resulted in a higher than average posterior probability in the marker intervals surrounding the QTL position, *i.e*. the QTL was often mapped in one of the neighbouring intervals (Fig. 3 and 4). Window sizes of 6 and 12 generally yielded the highest posterior probabilities in the marker interval where the QTL was simulated, while for the larger QTL the posterior probabilities in the surrounding intervals tended to be lower than those obtained with window sizes of 2 and 20 (Fig. 3). *LimitIBD *for the base haplotypes hardly influenced the posterior probabilities (results not shown).

## Discussion

### Haplotype clustering and window size

The aim of this paper was to investigate the effect of the number of surrounding marker alleles (window size) included in base haplotypes and the effect of clustering of base haplotypes based on IBD probabilities, on the accuracy of genomic breeding value prediction and QTL mapping. Window size had a strong effect on QTL mapping, where windows of six and 12 markers gave the best results, which was in agreement with the results of Grapes *et al*. [4] and to some extent with the results of Hayes *et al*. [19]. Hayes *et al*. [19] have found that including more marker alleles in haplotypes to evaluate a QTL position, leads to a higher proportion of the QTL variance being explained. However, it should be noted that in the study of Hayes *et al*., [19] the use of smaller haplotypes means that less marker alleles were used in the evaluation because only one putative QTL position was considered. In our application, smaller haplotypes implies that alleles of a certain marker are used to evaluate fewer putative QTL positions, but alleles of all markers are still used in the analysis. Grapes *et al*. [4] have reported that the predicted genomic breeding value of an animal is the same for haplotype sizes of four to ten markers, but lower for haplotypes of one marker. In comparison, at values for *limitIBD *of 0.75 and 0.95, we found the same accuracies for genomic breeding values at windows of six to 20 markers, and a slightly lower accuracy for windows of two markers.

Overall, the achieved accuracy in this study of 0.79 to 0.81 was comparable to values reported in other studies with similar marker densities [3,1,10].

Different window sizes and *limitIBD *values were only applied for the base haplotypes. Arguably, the approach of clustering base haplotypes based on the IBD-matrix is somewhat comparable to using genetic groups in a polygenic model. In both situations, individuals (haplotypes or animals) with incomplete relationships between them are clustered. IBD probabilities between non-base haplotypes are more 'complete' than those between base haplotypes, since their ancestral haplotypes are known. Analogous to the situation with genetic groups, where the need to group individuals decreases when the relationships become more complete [20], the different *limitIBD *values were not considered for the non-base haplotypes. Nevertheless, non-base haplotypes with an IBD probability > 0.95 were clustered in all cases, to reduce the number of (strongly related) effects.

Intuitively, the minimum requirement for any pair of haplotypes to be clustered is that the predicted chance that they are IBD is larger than the predicted chance that they are non-IBD. This implies that *limitIBD *should be at least larger than 0.50. Therefore, the lowest applied *limitIBD *value for clustering of haplotypes, 0.55, might appear to be rather extreme. However, the results show that such an extreme *limitIBD *value actually give similar results as the other values, while the number of haplotype effects that need to be estimated are reduced by at least 50%. A comparable strategy proposed by Ronnegard *et al*. [21] reduces the dimensions of the IBD matrix by using a submatrix of the IBD matrix selected based on the eigenvalues of the IBD matrix. This method also substantially reduces the rank of the IBD matrix, while the predicted QTL position is not affected.

Estimated total haplotype variance appeared to be more or less independent of *limitIBD *and window size, whereas estimated polygenic variance did appear to depend on these two parameters. When considering only the polygenic variances, it is expected that differences across *limitIBD *and window size are due to differences of explained genetic variance by the haplotype effects. A possible explanation for not finding a relation between the estimated haplotype variances and *limitIBD *or window size may be that the chosen assumptions when calculating the IBD matrix, *i.e*. that the base generation was 100 generations ago and that the effective population size was 100 across those generations, affected the estimated haplotype variances. To further investigate the relation between estimated haplotype variance and *limitIBD *and window size, we calculated per analysis the variance of the posterior total estimated breeding values (including polygenic effects) of the 2100 animals in the data, assuming no relations between animals. These estimates ranged from 0.36 to 0.38. After subtracting the estimated polygenic variance, to obtain a surrogate for the total haplotype variance, these estimates for the haplotype variance did complement the trends of the polygenic variances across windows sizes and values of *limitIBD*. Although this alternative method relies on the violated assumption that the 2100 animals in the analysis are unrelated, these additional results indicate that the applied method to estimate total haplotype variance directly from the estimated haplotype effects needs further verification.

### Genomic breeding value prediction versus QTL mapping

When comparing our results based on windows of two or six markers, one could conclude that the best model for genomic breeding value prediction is not necessarily the best model for QTL mapping. For both genomic breeding value prediction and QTL mapping, the aim is to accurately predict the effect of QTL alleles. The main difference is that genomic breeding value prediction aims at predicting total breeding values with high accuracy, while QTL mapping aims at predicting the position of a QTL correctly. Consider a situation as shown in Figures 3 and 4, where one QTL is surrounded by a number of markers. For QTL mapping, the aim is to maximize the contrast in explained variance by the marker interval where the QTL is located and the other marker intervals. For genomic breeding value prediction, the aim is to maximize the amount of the QTL variance that is captured by the haplotypes of the marker intervals. This suggests that models which fit the data best, *i.e*. explain most of the variance, may be the most optimal for genomic selection, while the most optimal models for QTL mapping may actually not have the best fit to the data.

The multiple QTL model that we have applied can detect QTL of reasonable size, as demonstrated by Meuwissen and Goddard [17]. For both the application of genomic selection and multiple QTL mapping, it is important that QTL that are located close together are actually identified as two different QTL to allow for selection of animals with different combinations of QTL alleles. Since the data varied from replicate to replicate in terms of the distance between and the size of QTL, a proper investigation of the ability to separate nearby QTL was not possible based on our results. However, Uleberg and Meuwissen [22] have shown that a multiple QTL model comparable to the model used in our study could distinguish two QTL located 15 cM apart.

## Conclusion

The applied model, which considers all putative QTL positions simultaneously, has proven to be useful both for predicting total breeding values based on genome-wide markers and for QTL mapping. Intermediate window size led to more precise QTL mapping while increasing window size and decreasing clustering limit strongly reduced the number of haplotypes. Thus, we conclude that different optimal window sizes should be used in QTL-mapping versus genome-wide breeding value prediction.

## Competing interests

The authors declare that they have no competing interests.

## Authors' contributions

MPLC further developed the programs used for analysis, carried out the simulations and analyses, and wrote the first draft of the paper. RFV supervised the research and mentored MPLC. THEM developed initial versions of the programs used for analysis. THEM, JJW, EFK, CS and ALJV took part in useful discussions and advised on the analyses. All authors read and approved the final manuscript.
